# Maternal and Neonatal Outcomes in Preeclampsia With and Without Proteinuria

**DOI:** 10.7759/cureus.101534

**Published:** 2026-01-14

**Authors:** Chaiti Saha, Shikha Chadha, Neha Agrawal, Hima Chandana Kille, Puneeta Mahajan

**Affiliations:** 1 Obstetrics and Gynaecology, Dr. Baba Saheb Ambedkar Medical College and Hospital, Delhi, IND

**Keywords:** endorgan dysfunstion, maternal and fetal outcome, preeclampsia, preeclampsia complications, proteinuria, severe preeclampsia

## Abstract

Objective

Preeclampsia is the commonest medical complication of pregnancy and can be associated with significant maternal as well as neonatal morbidity and mortality. Proteinuria is the most consistent finding of preeclampsia, though it's not a must, according to the latest guidelines. However, proteinuria is considered one of the features of preeclampsia, and it is associated with adverse pregnancy outcomes. The role of proteinuria remains controversial as a prognostic marker due to variable correlation with severity; it may appear late in renal involvement. Also, gestational age and other end-organ involvement may influence the management decisions. This comparative study aims to examine whether the presence of proteinuria correlates with poorer maternal and neonatal outcomes.

Material and methods

A prospective observational study was conducted at a referral hospital. Six hundred women with preeclampsia after 24 weeks of gestation were included in the study, 300 in each group A & B, that is, preeclampsia with & without proteinuria, respectively. Proteinuria was assessed using a urine dipstick value of 2+ or more. All women were evaluated for end-organ involvement or uteroplacental insufficiency. Women were treated according to hospital protocol. Maternal and neonatal outcomes were studied and statistically analyzed. Quantitative variables were compared using an unpaired t-test. Qualitative variable has been compared using the Chi-square test /Fisher’s exact test.

Results

Overall maternal complications, where all complications were weighted equally, were significantly higher in Group A (96.3% vs 70.3%, p<0.0001).With a higher incidence of eclampsia (p=0.012), abruptio placenta (p=0.002), and hemolysis, elevated liver enzymes, and low platelet (HELLP) syndrome. Renal, hepatic, and hematological dysfunctions were more prevalent in Group B, posing serious challenges. Group A had a significantly higher percentage of overall neonatal complications: 44.6% vs 23.9% (p<0.0001), stillbirths (7.67% vs 1.67%), and intrauterine growth restrictions (IUGR) (12.33% vs 3.33%). Group B had higher rates of intrauterine deaths (IUD) (5.33% vs 0%), and neonatal intensive care unit (NICU) admissions were higher in Group B (p<0.0001). Thus, proteinuria appears to be a strong marker of poor neonatal health.

Conclusion

The study emphasizes that proteinuria, while important, is not the single most important factor in determining maternal and neonatal outcomes. Maternal and fetal outcomes also depend on other clinical factors, including systemic organ involvement and maternal comorbidities. This study affirms that women with proteinuria showed more neonatal and obstetric maternal complications, but women with non-proteinuric preeclampsia can still be associated with significant biochemical derangements and pose difficulties in management.

## Introduction

Preeclampsia [PE] is a complex multiorgan disorder with multifactorial etiology. It is the commonest medical complication of pregnancy affecting 3-10% of pregnant women [1]. It is often associated with maternal and fetal morbidity and mortality. Traditionally, proteinuria has been the hallmark of diagnosing preeclampsia. However, the 2018 guideline issued by ISSHP has defined preeclampsia as hypertension associated with either proteinuria, multiorgan dysfunction, or uteroplacental insufficiency [2]. Different International bodies like the American College of Obstetricians and Gynecologists (ACOG), National Institute for Health and Care Excellence (NICE), and the Society of Obstetric Medicine of Australia and New Zealand (SOMANZ) guidelines for management of hypertensive disorders in pregnancy also accepted that proteinuria is not a mandatory factor in the diagnosis of PE, but played an important role in assessing the prognosis of PE [3-5]. At the same time, some scientists still hold the opinion that proteinuria is closely related to adverse pregnancy outcomes. Proteinuria is the most consistent finding of preeclampsia, though it's not a must according to the latest guidelines. 

There was a long debate as to the importance of proteinuria in diagnosing preeclampsia. Excluding the mandatory proteinuria from diagnostic criteria would add a greater number of women to the preeclampsia category. Therefore, these women will be subjected to detailed investigations and monitoring, leading to economic burden and psychological consequences. On the other hand, missing out on some women with preeclampsia can cause significant adverse outcomes for them.

The main pathology behind this preeclampsia is abnormal placentation, which results in the release of angiogenic factors that cause inflammation, vasoconstriction, endothelial disruption, and increased vascular permeability. Proteinuria reflects endothelial damage in the glomeruli, which causes leakage of protein into urine. Textbooks typically write that 10% of women with preeclampsia and 20% of women with eclampsia may present without proteinuria despite having clinical symptoms. The reason may lie in the fact that the organ dysfunctions involving the liver and kidney can still be present in the absence of proteinuria, and thus, the gravity of the disease may not become apparent. 

The role of proteinuria remains controversial as a prognostic marker due to variable correlation with severity; it may appear late in renal involvement. Also, gestational age and other end-organ involvement may influence the management decision. Many studies have reported poor perinatal outcomes when proteinuria is present, while others have reported poor outcomes even in the absence of proteinuria [6]. It has been found that isolated proteinuria without preeclampsia is also associated with adverse pregnancy outcomes [7,8]. The study was conducted to compare maternal and neonatal outcomes in preeclamptic women with and without proteinuria among all preeclamptic women of more than 24 weeks of gestation.

## Materials and methods

Study design and setting

A prospective observational study was conducted at a tertiary hospital in Delhi over a period of 20 months from 1st December 2019 to 31st July 2021. The study was approved by the Institutional Ethics and Research Board of the Dr. Baba Saheb Ambedkar Medical College with approval number F5(59)2017/BSAH/DNB/29982 dated November 28, 2019. Written informed consent was obtained from each patient in their native language before enrolment.

Sample size

A study conducted by Tochio et al. found an odds ratio of cesarean delivery, hemolysis, elevated liver enzymes, low platelet (HELLP) syndrome, and appearance, pulse, grimace, activity, respiration (APGAR) score <7, admission to NICU of 0.66, 0.47, 1.85, and 0.77, respectively [9]. Taking these values as reference, the minimum required sample size with 99% power of the study and two-sided alpha of 1% was 219 patients per group. To reduce the margin of error, a sample size of 300 is used in each group.

Formula used is "$&quot; n \ge \frac{2\left(Z_{\alpha} + Z_{\beta}\right)^2}{\mathrm{OR}^2} &quot;$"

 where Z α is the value of Z at a two-sided alpha error of 1% and, Z β is the value of Z at a power of 99%, and OR is the odds ratio.

Inclusion and exclusion criteria

Women with singleton live pregnancy with preeclampsia were included in the study. Diagnosis of preeclampsia was made based on the International Society for the Study of Hypertension in Pregnancy (ISSHP) guideline 2018. Women with multiple pregnancies, fetal abnormalities, chronic renal disease, chronic liver disease, and autoimmune disease were excluded from the study.

Methodology

All pregnant women admitted with a diagnosis of preeclampsia after 24 weeks of gestation in the department of obstetrics and gynecology were screened for inclusion and exclusion criteria. Those eligible for the study were recruited after taking informed consent. A detailed evaluation was done, which included a thorough history, general physical, systemic, and obstetrical examination. Gestational age was confirmed by the last menstrual period (LMP) or first-trimester ultrasound. A note regarding right upper quadrant pain or epigastric pain, altered mental status, blindness, clonus, severe intractable headache, and visual scotomata was made. Antenatal care (ANC) routine investigations were reviewed. Blood pressure was recorded after 5 min of rest, with an automatic electronic standardized device with an appropriately sized cuff. We took two readings four hours apart before labelling women as hypertensive. Proteinuria was checked for with the help of the urine dipstick method, and a value of 2+, which is 100 mg/dL, was taken as proteinuria positive. Urine dipstick testing was not repeated. Protein/creatinine ratio or 24-hour urine was available for borderline cases. Those who were having proteinuria were included in group A, and those without proteinuria were included in group B. Investigations were done to find out end-organ involvement or uteroplacental insufficiency. These comprised complete blood count with peripheral smear, liver and kidney function tests, lactate dehydrogenase (LDH) (if HELLP was suspected), coagulation profile, ultrasound of the kidneys, obstetrics ultrasound, and Doppler study. Uteroplacental insufficiency was defined based on Doppler evidence of impaired placental perfusion, including elevated uterine artery and/or umbilical artery pulsatility index (>95th percentile for gestational age), persistent uterine artery notching beyond 24 weeks, reduced or absent end diastolic flow in the umbilical artery, and/or a reduced cerebroplacental ratio(<10th percentile).

Patient management was done as per hospital protocol, which encompassed monitoring of the patient, antihypertensive drug use with systolic blood pressure more than 150 mm Hg and diastolic blood pressure more than 100 mm Hg, and anticonvulsive drug (Pritchard regime of magnesium sulphate). Monitoring and supervision were continued till 37 weeks or till an indication for termination of pregnancy developed or the patient went into labor. Patients were followed up for seven days post-delivery. All antepartum, intrapartum, and postpartum events were recorded. Neonatal outcomes were also recorded. Outcome assessors were not blinded to proteinuria status, but they simply noted the complications from the progress notes, which were written by different managing teams for each case.

Maternal outcomes recorded were pulmonary oedema, retinal detachment, eclampsia, HELLP syndrome, disseminated intravascular coagulation, abruptio, post-partum hemorrhage, preterm labor, mode of delivery, etc. Neonatal outcomes were noted, such as prematurity, IUGR, NICU admission rate, stillbirth, and neonatal deaths. Maternal and perinatal outcomes in the two groups were compared separately and also in a combined composite manner, where all complications were given equal weightage, and statistical analysis was done.

Statistical analysis

The categorical variables are presented in numbers and percentage and the continuous variables are presented as mean ±SD and median. Quantitative variables were compared using Unpaired t-test between the two groups. Qualitative variables were compared using the Chi-square test. A p value of less than 0.05 was considered statistically significant.

## Results

We screened 628 women with preeclampsia. Twenty-eight women were excluded from the study, 26 due to multiple pregnancy, and two had chronic renal disease. Six hundred women were enrolled for the study, 300 in each group A & B, that is, preeclampsia with & without proteinuria, respectively. Demographic data of the study population is depicted in Table 1, and it shows no significant difference between the two groups. The mean age of our study group was 24.32±3.26.

**Table 1:** Sociodemographic characteristics of two study groups Age is presented as mean ±standard deviation, other values are presented as N(%). § Independent t test, † Fisher's exact test, ‡ Chi-square test, * p value <0.05.

Sociodemographic characteristic	Group A (n=300)	Group B (n=300)	Total	p value	Test performed
Age (years) Mean±SD	24.51±3.29	24.14±3.22	24.32±3.26	0.164§	t-test;1.392
Education					
Illiterate	12 (4%)	20 (6.67%)	32 (5.33%)	0.116†	Fisher's exact test
Primary	76 (25.33%)	71 (23.67%)	147 (24.50%)		
Secondary	146 (48.67%)	123 (41%)	269 (44.83%)		
Senior secondary	61 (20.33%)	82 (27.33%)	143 (23.83%)		
Graduate	5 (1.67%)	4 (1.33%)	9 (1.50%)		
Occupation					
House wife	300 (100%)	300 (100%)	600 (100%)	-	-
Socio-economic status					
Upper middle	20 (6.67%)	20 (6.67%)	40 (6.67%)	0.854‡	Chi-square test,0.782
Lower middle	110 (36.67%)	120 (40%)	230 (38.33%)		
Upper lower	21 (7%)	21 (7%)	42 (7%)		
Lower	149 (49.67%)	139 (46.33%)	288 (48%)		
Religion					
Hindu	290 (96.67%)	283 (94.33%)	573 (95.50%)	0.168‡	Chi-square test,1.9
Muslim	10 (3.33%)	17 (5.67%)	27 (4.50%)		
Parity					
Nullipara	198 (66%)	227 (75.67%)	425 (70.83%)	0.029‡*	Chi-square test,7.078
Primi para	67 (22.33%)	45 (15%)	112 (18.67%)		
Multipara	35 (11.67%)	28 (9.33%)	63 (10.50%)		

As expected, most of the patients were primigravidae in the study, i.e., 339 (70.83%). No significant differences were observed in either the history (p=0.731) or family history (p=1.0) of preeclampsia.

The mean period of gestation at enrollment in group A was 36.6±2.74 weeks, and in group B was 36.6±2.81 weeks. The range of gestational age at recruitment in the study was 25-41 weeks in group A and 26-41weeks in group B. Preeclampsia was detected in 266 women(55.5%) of more than 37 weeks of gestation (136 (56..66%) & 130 (54.17%) in group A & B, respectively). In 136 (28%) women, preeclampsia was detected at late preterm gestation, that is, 34-37 weeks of gestation (70(29.17%) and 66(27.5%) in group A and group B, respectively). About 69 cases (14%) were detected at <34 weeks & only nine cases(1.8%) were detected at a gestation of less than 28 weeks.

Signs and symptoms of imminent eclampsia were reported by 51 (17 %) women in group A & 64 (21.3 %) women in group B. (p=0.003). This emphasizes that proteinuria preeclampsia may present with more overt clinical signs. 

When women’s body mass index (BMI) was compared in two groups, it was found that 17(7%) women were overweight and 16 women(6.67%) were obese in group A, whereas in group B, three women(12.50%) were overweight and 24 women(10%) were obese. The difference was not statistically significant. There was no significant difference between blood pressure in the two groups of women at presentation. The mean systolic blood pressure in group A was 157.77±13.71 mmHg, and in group B, 157.84±12.47mmHg and the mean diastolic blood pressure was 103.04±13.05 and 101.47 mmHg±10.03, respectively.

The distribution of involvement of different organs between the groups is shown in Table 2. The difference between the two groups was significant, p value <0.05, for the frequency of thrombocytopenia and abnormal renal function test.

**Table 2:** End organ involvement in two study group Values are presented as N(%). † Fisher's exact test, ‡ Chi-square test, * p value <0.05.

Organ dysfunction	Group A (n=300)	Group B (n=300)	Total	p value	Test performed
Liver SGOT/SGPT >2	78 (26.00%)	88 (29.33%)	166 (27.67%)	0.361‡	Chi-square test,0.833
Kidney Creatinine >1.1	13 (4.33%)	42 (14.0%)	55 (9.17%)	<0.0001‡*	Chi-square test,16.834
Thrombocytopenia <1 lac	32 (10.67%)	68 (22.67%)	100 (16.67%)	<0.0001	Chi-square test,15.552
Abnormal doppler	57 (19%)	69 (23.0%)	126 (21.00%)	0.229‡	Chi-square test,1.447
Cerebral symptoms	28 (9.33%)	10 (3.33%)	38 (6.33%)	0.003‡*	Chi-square test,9.103
Visual symptoms	28 (9.33%)	10 (3.33%)	38 (6.33%)	0.003‡*	Chi-square test,9.103
Pulmonary edema	03 (1.0%)	14 (4.67%)	17 (2.83%)	0.011†*	Fisher's exact test

The incidence of maternal complications and comparison between the two groups is shown in Table 3. On comparing the two groups concerning these, only abruptio placenta reached statistically significant levels. When composite maternal complications were compared, where all complications were weighted equally, the difference was found to be statistically significant with a p value <0.0001 and an odds ratio of 0.094 (0.049-0.178). The use of antihypertensive and anticonvulsive drugs was higher, apparently due to a greater incidence of impending features and development of eclampsia. Postpartum hemorrhage was significantly higher in group B.

**Table 3:** Maternal complications Values are presented as N (%). † Fisher's exact test, ‡ Chi-square test, * p value <0.05.

Complications	Group A (n=300)	Group B (n=300)	Total	p value	Test performed
Antepartum					
Eclampsia	12 (4%)	2 (0.67%)	14 (2.33%)	0.012†*	Fisher's exact test
HELLP	10 (3.33%)	3 (1%)	13 (2.17%)	0.089†	Fisher's exact test
Abruptio placenta	10 (3.33%)	0 (0%)	10 (1.67%)	0.002†*	Fisher's exact test
Retinal abnormalities	0 (0%)	1 (0.33%)	1 (0.17%)	1.000†	Fisher's exact test
Intrapartum					
Eclampsia	15 (5%)	20 (6.67%)	35 (5.83%)	0.384‡	Chi-square test,0.759
Abruptio placenta	11 (3.67%)	5 (1.67%)	16 (2.67%)	0.128‡	Chi-square test,2.312
Postpartum					
Eclampsia	2 (0.67%)	4 (1.33%)	6 (1%)	0.686†	Fisher's exact test
Shock	0 (0%)	1 (0.33%)	1 (0.17%)	1.000†	Fisher's exact test
Pulmonary edema	2 (0.67%)	7 (2.33%)	9 (1.50%)	0.176†	Fisher's exact test
Maternal death	14 (4.67%)	7 (2.33%)	21 (3.50%)	0.120‡	Chi-square test,2.418
Postpartum haemorrhage	55(18.33%)	44(14.67%)	99(16.50%)	0.226‡	Chi-square test,1.464

The mean period of gestation to delivery in group A was 37.47±2.12 weeks, and in group B was 37.27±2.34 weeks, p value <0.272. Preterm delivery rate was higher in group B, 90(37.33%), as compared to group A, 76(31.67%), p value <0.144.

The mode of delivery was similar in both groups, as shown in Table 4. Overall caesarean section rate was 82(20.87%) in full-term pregnant women as compared to 50(24.15 %) in the preterm preeclamptic women. Overall, no statistically significant difference was found between the two groups.

**Table 4:** Mode of delivery Values are presented as N(%). † Fisher's exact test, ‡ Chi-square test.

	Mode of delivery	Group A (n=300)	Group B (n=300)	Total	p value	Test performed
Term	Vaginal delivery	161 (78.54%)	150 (79.79%)	311 (79.13%)	0.761‡	Chi-square test,0.093
	LSCS	44 (21.46%)	38 (20.21%)	82 (20.87%)		
Preterm	Vaginal delivery	71 (74.74%)	86 (76.79%)	157 (75.85%)	0.731‡	Chi-square test,0.118
	LSCS	24 (25.26%)	26 (23.21%)	50 (24.15%)		

On comparing composite fetal outcome, the difference between the two groups was statistically significant. Prematurity, meconium-stained liquor, and IUD were higher in group B. Whereas intra-uterine growth retardation and stillbirth were higher in group A. Proteinuria thus seems to be associated more with a compromised fetus. Whereas, acute fetal compromise and prematurity were associated more with the organ dysfunction.

In our study Neonatal outcomes were significantly different between the two groups, with better outcomes seen in group B for NICU admission and still births (Table 5). This again points toward the fact that proteinuria led to poor outcome in the form of a low-birth-weight baby, with an appearance, pulse, grimace, activity, respiration (APGAR) score less than 7 at five minutes, for similar outcomes in both groups. NICU admissions were higher in group A (statistically significant), while neonatal deaths were insignificantly higher. There was no difference in the proportion of maternal complications with respect to the period of gestation in groups A and B.

**Table 5:** Neonatal complications Values are presented as N(%). † Fisher's exact test, ‡ Chi-square test, * p value <0.05. NICU: neonatal intensive care unit, RDS: respiratory distress syndrome, FGR: fetal growth. restriction

Neonatal complications	Group A (n=300)	Group B (n=300)	Total	p value	Test performed
NICU	104 (34.67%)	45 (15.85%)	149 (25.51%)	<0.0001‡*	Chi-square test,31.081
Stillbirth	23 (7.67%)	05 (1.76%)	28 (4.79%)	0.0008‡*	Chi-square test,12.138
FGR	41 (13.67%)	26 (9.15%)	67 (11.47%)	0.052‡	Chi-square test,3.780
RDS	19 (6.33%)	22 (7.75%)	41 (7.02%)	0.627‡	Chi-square test,0.236
Neonatal death	11 (3.67%)	3 (1.06%)	14 (2.40%)	0.054†	Fisher's exact test

## Discussion

One of the hallmark features of preeclampsia was the presence of proteinuria. While modern diagnostic criteria have evolved to recognize other manifestations of systemic involvement beyond proteinuria, the detection of protein in the urine remains a vital clinical indicator. The effect of proteinuria on maternal and perinatal outcomes is being investigated thoroughly. Its role in diagnosis, monitoring, and development of long-term complications is also of significant interest. Researchers have proposed that although proteinuria is an important predictor of poor maternal and neonatal outcomes, it is only one of the contributors [10]. The presence of inflammatory markers is said to provide more pointed prognosticators of maternal complications like eclampsia, HELLP, and renal dysfunction.

Tamas et al. recently proposed to classify preeclampsia into early onset proteinuria “placental preeclampsia” and late onset non-proteinuria “maternal preeclampsia” [11]. They report more adverse fetal outcomes in placental type and greater maternal complications in maternal type, often presenting without proteinuria but marked by endothelial damage, microangiopathy, and renal compromise. This hypothesis of two distinct pathophysiological pathways was further evidenced in murine models by Wei et al. and has profound implications for diagnosis, monitoring, and managing preeclampsia [12].

Various studies have shown that maternal and perinatal outcomes are poor with higher levels of proteinuria. It has been found that severe proteinuria led to the onset of preeclampsia and delivery at an earlier gestational age, as well as a higher incidence of fetal growth restriction (FGR) [13]. In a retrospective study, it was established that massive proteinuria results in early-onset preeclampsia and preterm delivery [10]. Also, these women had more severe clinical presentations and a higher risk of abnormal lab values. The perinatal outcomes, including fetal growth restriction (FGR), preterm delivery, stillbirth, and neonatal complications, are found to be associated with the amount of proteinuria.

In our study, proteinuria patients demonstrated a significantly higher frequency of symptoms suggesting impending eclampsia, such as headache, visual disturbances, and epigastric pain. This supports the literature that proteinuria PE tends to present more symptomatically, possibly due to more overt endothelial involvement [11]. This supports the classical view that proteinuria in preeclampsia is more symptomatic, possibly due to more overt endothelial dysfunction. Moreover, Group A required significantly more antihypertensive (96.3%) and anticonvulsant therapy (6%), suggesting a more aggressive clinical course. In contrast, a significant proportion of women without proteinuria were asymptomatic, similarly reported by Biju and Backer, who highlighted the diagnostic challenges posed by “silent” non-proteinuria preeclampsia [14]. Despite the paucity of symptoms, these women exhibited substantial biochemical abnormalities, raising concerns about delayed diagnosis and underestimation of disease severity in such cases. Tanacan et al. have reported elevated markers of maternal organ dysfunction, including liver enzymes and serum uric acid, in non-proteinuria cases [15].

As far as maternal outcomes are concerned, women in group A had a significantly higher incidence of eclampsia, abruptio, and HELLP. Admission to ICU, although higher in this group were not statistically significant. When overall maternal complications were compared, Group A women were found to be more susceptible to maternal complications with an Odds ratio of 0.094. The proteinuria was thus found to be associated with worsening of preeclampsia and its complications. On comparing the fetal outcomes, women in the proteinuria group had significantly higher rates of stillbirth, FGR, and NICU admissions. Overall, neonatal complications and mortality were significantly higher in the proteinuria group, indicating that proteinuria may be a marker for worse neonatal outcome (p<0.0001). It is important to note that these associations were found in our observational study and therefore do not imply any causality. These results align with the study by A. Tanacan et al., which found a significantly higher composite maternal adverse outcome in proteinuric women (79.2%) versus 6.9% in non-proteinuric cases, and they also found SGA and LBW to be more common in proteinuric preeclamptic women [15]. Chikizie et al. reported that women have a greater incidence of placental insufficiency, IUGR, oligohydramnios, prematurity, and stillbirth [16]. Lei et al. in their study reported stillbirths in 17% of proteinuric vs. none in non-proteinuric cases [17].

This supports the theory that proteinuria correlates with impaired placental function, leading to fetal hypoxia, growth restriction, and higher perinatal mortality. Conversely, non-proteinuric PE may represent a more maternal-driven pathology, where fetal effects are less pronounced, but maternal organ involvement may be more insidious.

The current study found significantly higher thrombocytopenia and renal dysfunction (elevated creatinine), suggesting non-proteinuric PE may present with more severe organ dysfunction despite being clinically less overt. Liver function derangements, while not statistically different, were noted in both groups. These findings mirror those reported by a few authors, who found deranged renal and hematological parameters even in patients without proteinuria, highlighting that proteinuria does not reliably reflect systemic disease severity. [15,17]. The presence of proteinuria does not point to the full extent of microangiopathy, particularly affecting the hematological and renal systems. These results underscore the need to incorporate objective biochemical and hematological assessment of all preeclamptic patients, regardless of urinary protein status.

Group B also presented with higher postpartum pulmonary oedema (2.33%) and ICU admissions, although these were not statistically significant. However, it is noteworthy that Group B was not devoid of risk. Maternal morbidity was still high (70.33%), with cases of pulmonary edema, ICU admissions, and postpartum complications such as hemorrhage and seizure. Our findings echo those of Tochio et al., who observed comparable maternal composite outcomes in both groups [9]. End-organ damage and life-threatening complications like abruptio and eclampsia can result in poor maternal outcomes [14]. The implication is clear that the absence of proteinuria should not equate to a lower-risk designation, and all hypertensive pregnancies warrant vigilant monitoring.

Interestingly, preterm delivery was slightly more frequent in Group B, suggesting that although the proteinuric PE group had worse neonatal outcomes, the non-proteinuric PE may trigger earlier medical interventions due to sudden maternal deterioration (e.g., thrombocytopenia or rising creatinine). Proteinuric PE demonstrated a significantly higher burden of placental insufficiency-related outcomes, such as placental abruption and stillbirth. Fetal growth restriction was also more frequent in this group, though not statistically significant. NICU admission was higher in group A, likely reflecting earlier delivery or intensified clinical management rather than intrinsic neonatal morbidity. Rates of respiratory distress syndrome and neonatal death were comparable, suggesting outcomes were primarily mediated by prematurity rather than placental failure.

Thus, in our study, end-organ involvement did influence maternal outcome irrespective of proteinuria. This further underscores the importance of distinguishing between maternal and placental phenotypes of preeclampsia, as proposed by Tamas et al. [11].

While proteinuria remains a marker for increased neonatal risk and placental pathology, it does not capture the full clinical spectrum of preeclampsia. Non-proteinuric preeclampsia is associated with significant maternal morbidity, particularly renal and hematological dysfunction, despite its often asymptomatic presentation.

The findings from this study highlight the inadequacy of proteinuria as a sole marker for preeclampsia severity. While it correlates with increased maternal and neonatal complications due to placental insufficiency, its absence does not exclude the presence of significant maternal morbidity. Other authors have similarly highlighted the clinical subtlety of non-proteinuric preeclampsia and its potential for underdiagnosis [11,18]. The muted symptom profile in non-proteinuric patients emphasizes the need for routine systemic screening of all hypertensive pregnancies beyond urinalysis alone.

Absence of proteinuria may sometimes result in delayed interventions and is also found to be associated with adverse fetal outcome [14]. Other researchers reported a higher incidence of fetal distress, poor APGAR score [19, 20].

In clinical practice, many clinicians use proteinuria levels to make clinical decisions regarding the delivery of preeclampsia cases. The increased levels of proteinuria are considered to worsen the progression of preeclampsia and result in poor perinatal outcomes.

Preeclampsia remains one of the most challenging issues in obstetrics, which causes maternal and perinatal morbidity as well as mortality. It is well accepted that the timely and correct prediction of preeclampsia can help identify pregnant women at greater risk for various complications. This will help obstetricians to monitor these patients with more vigilance to achieve better pregnancy outcomes. However, the pregnancy outcome of patients with preeclampsia is affected by many factors, so the monitoring of patients who present with proteinuria combined with the other presenting features and other laboratory indicators may guide the management better in order to avoid adverse pregnancy outcomes.

Emerging studies on the influence of proteinuria in women with preeclampsia suggest that the outcome may not differ in women with and without proteinuria [21]. Preeclampsia with proteinuria is set to be associated with a greater incidence of progression to severe preeclampsia and causing end organ dysfunction. Presence of proteinuria correlates more strongly with poorer fetal and neonatal outcomes and increased risk of fetal growth restriction, preterm birth, and perinatal mortality. The placenta of women with proteinuria reveals more pronounced abnormalities.

The present study and corroborating literature confirm that preeclampsia with and without proteinuria represent distinct clinical entities, with overlapping but divergent maternal and fetal consequences. While proteinuria remains a marker of placental pathology and fetal risk, its absence does not confer immunity from severe maternal morbidity. A comprehensive, biomarker-augmented, phenotype-aware approach is warranted to optimize care and reduce the adverse outcomes of this multifaceted disorder.

Our study has a good number of patients, but has certain limitations. It’s a single-center study. Another limitation of the study is that some women in the proteinuria group also had organ dysfunction. This was a confounding factor for the results. We did not analyze this group separately. We relied on urine dipstick testing for categorizing significant proteinuria. Diagnosis of proteinuria based on urine protein dipstick is comparatively a crude method but is accepted by ISSHP, and its sensitivity is said to be better when 2+ or more is used, but potential misclassification is possible since hydration, timing, and urine concentration can affect results. This study did not consider ruling out all the compounding factors for fetal growth restrictions, and thus, FGR can only be said to be associated with and not as a result of preeclampsia. One should keep in mind that the severity of underlying placental dysfunction was not directly quantified and may have acted as a confounding factor influencing both proteinuria status and feto-maternal outcomes. The study was conducted in a tertiary referral center, where referral patterns and disease severity may differ from lower- level care settings. Therefore, the findings may have limited generalizability to non-referral or lower-risk populations.

## Conclusions

The study emphasizes that although proteinuria has traditionally been considered a central feature of preeclampsia, it is not the sole determinant of maternal and neonatal outcomes. Adverse outcomes are influenced by a combination of factors, including the degree of systemic organ involvement, severity of hypertension, and pre-existing maternal comorbidities. In this study, women with proteinuria demonstrated higher rates of neonatal and obstetric complications, reaffirming proteinuria as an important marker of disease severity. However, non proteinuric preeclampsia can still be associated with significant biochemical derangements presenting diagnostic and management challenges, underscoring the need for a comprehensive clinical assessment beyond proteinuria alone.

## References

[1] Cunningham F, Leveno KJ, Bloom SL (2018). Obstetrical complications: hypertensive disorders. Williams Obstetrics.

[2] (2013). Hypertension in pregnancy. Report of the American College of Obstetricians and Gynecologists’ Task Force on Hypertension in Pregnancy. Obstet Gynecol.

[3] Tranquilli AL, Dekker G, Magee L (2014). The classification, diagnosis and management of the hypertensive disorders of pregnancy: a revised statement from the ISSHP. Pregnancy Hypertens.

[4] Brown MA, Magee LA, Kenny LC (2018). Hypertensive Disorders of Pregnancy: ISSHP Classification, Diagnosis, and Management Recommendations for International Practice. Hypertension.

[5] Lowe SA, Bowyer L, Lust K (2015). SOMANZ guidelines for the management of hypertensive disorders of pregnancy 2014. Aust N Z J Obstet Gynaecol.

[6] Bramham K, Poli-de-Figueiredo CE, Seed PT, Briley AL, Poston L, Shennan AH, Chappell LC (2013). Association of proteinuria threshold in pre-eclampsia with maternal and perinatal outcomes: a nested case control cohort of high risk women. PLoS One.

[7] Bae EH, Kim JW, Choi HS, Ma SK, Kim SW (2017). Impact of random urine proteinuria on maternal and fetal outcomes of pregnancy: a retrospective case-control study. Korean J Intern Med.

[8] Kuyucu M, Arnikan Sevcan Arzu, Herkiloğlu Dilsad, Muhcu Murat Assessment of maternal and perinatal outcome in pregnant women with isolated proteinuria. Perinatal Journal2016.

[9] Tochio A, Obata S, Saigusa Y, Shindo R, Miyagi E, Aoki S (2019). Does pre-eclampsia without proteinuria lead to different pregnancy outcomes than pre-eclampsia with proteinuria?. J Obstet Gynaecol Res.

[10] Kim MJ, Kim YN, Jung EJ (2017). Is massive proteinuria associated with maternal and fetal morbidities in preeclampsia?. Obstet Gynecol Sci.

[11] Tamas P, Farkas B, Betlehem J (2025). Practical considerations concerning preeclampsia subgroups. J Clin Med.

[12] Wei Y, Tian H, Wei X (2024). Distinct phenotypes in the preeclamptic-like mouse model induced by adenovirus carrying sFlt1 and recombinant sFlt1 protein. Eur J Med Res.

[13] Dong X, Gou W, Li C, Wu M, Han Z, Li X, Chen Q (2017). Proteinuria in preeclampsia: not essential to diagnosis but related to disease severity and fetal outcomes. Pregnancy Hypertens.

[14] Biju N, Backer C (2025). Hidden preeclampsia leading to placental abruption and disseminated intravascular coagulation. Cureus.

[15] Tanacan A, Fadiloglu E, Beksac MS (2019). The importance of proteinuria in preeclampsia and its predictive role in maternal and neonatal outcomes. Hypertens Pregnancy.

[16] Chikezie K, Uche CL, Ocheni S (2025). Preeclampsia and its clinico-laboratory markers: a comparative study among pregnant women in Enugu State, Nigeria. Journal of Clinical and Laboratory Research.

[17] Lei T, Qiu T, Liao W (2021). Proteinuria may be an indicator of adverse pregnancy outcomes in patients with preeclampsia: a retrospective study. Reprod Biol Endocrinol.

[18] Vennela M, Yeddala M, Lakshmipriya Y (2025). A study of maternal and perinatal outcomes in severe preeclampsia and eclampsia in a tertiary care hospital. EJCM.

[19] Nakanishi S, Aoki S, Nagashima A, Seki K (2017). Incidence and pregnancy outcomes of superimposed preeclampsia with or without proteinuria among women with chronic hypertension. Pregnancy Hypertens.

[20] Owaraganise A, Migisha R, Ssalongo WG (2021). Nonproteinuric preeclampsia among women with hypertensive disorders of pregnancy at a referral hospital in Southwestern Uganda. Obstet Gynecol Int.

[21] Jose Oliveira Da Costa, Natalia Marchao, Nadiesda Peres (2024). Pregnancy in patients with chronic kidney disease stage 3 to 5:what to expect?. Nephrology Dialysis Transplantation.

